# The Effectiveness of a Digital App for Reduction of Clinical Symptoms in Individuals With Panic Disorder: Randomized Controlled Trial

**DOI:** 10.2196/51428

**Published:** 2024-04-12

**Authors:** KunJung Kim, Hyunchan Hwang, Sujin Bae, Sun Mi Kim, Doug Hyun Han

**Affiliations:** 1 Chung Ang University Hospital Seoul Republic of Korea

**Keywords:** digital app, mHealth, mobile health, app, apps, application, applications, functional near-infrared spectroscopy, hemodynamic, hemodynamics, panic disorder, anxiety, panic, mental, fear, spectroscopy, digital therapy, fNIRS, brain, imaging, neurology, neuroscience, cortex, cortices

## Abstract

**Background:**

Panic disorder is a common and important disease in clinical practice that decreases individual productivity and increases health care use. Treatments comprise medication and cognitive behavioral therapy. However, adverse medication effects and poor treatment compliance mean new therapeutic models are needed.

**Objective:**

We hypothesized that digital therapy for panic disorder may improve panic disorder symptoms and that treatment response would be associated with brain activity changes assessed with functional near-infrared spectroscopy (fNIRS).

**Methods:**

Individuals (n=50) with a history of panic attacks were recruited. Symptoms were assessed before and after the use of an app for panic disorder, which in this study was a smartphone-based app for treating the clinical symptoms of panic disorder, panic symptoms, depressive symptoms, and anxiety. The hemodynamics in the frontal cortex during the resting state were measured via fNIRS. The app had 4 parts: diary, education, quest, and serious games. The study trial was approved by the institutional review board of Chung-Ang University Hospital (1041078-202112-HR-349-01) and written informed consent was obtained from all participants.

**Results:**

The number of participants with improved panic symptoms in the app use group (20/25, 80%) was greater than that in the control group (6/21, 29%; *χ*^2^_1_=12.3; *P*=.005). During treatment, the improvement in the Panic Disorder Severity Scale (PDSS) score in the app use group was greater than that in the control group (*F*_1,44_=7.03; *P*=.01). In the app use group, the total PDSS score declined by 42.5% (mean score 14.3, SD 6.5 at baseline and mean score 7.2, SD 3.6 after the intervention), whereas the PDSS score declined by 14.6% in the control group (mean score 12.4, SD 5.2 at baseline and mean score 9.8, SD 7.9 after the intervention). There were no significant differences in accumulated oxygenated hemoglobin (accHbO_2_) at baseline between the app use and control groups. During treatment, the reduction in accHbO_2_ in the right ventrolateral prefrontal cortex (VLPFC; *F*_1,44_=8.22; *P*=.006) and the right orbitofrontal cortex (OFC; *F*_1,44_=8.88; *P*=.005) was greater in the app use than the control group.

**Conclusions:**

Apps for panic disorder should effectively reduce symptoms and VLPFC and OFC brain activity in patients with panic disorder. The improvement of panic disorder symptoms was positively correlated with decreased VLPFC and OFC brain activity in the resting state.

**Trial Registration:**

Clinical Research Information Service KCT0007280; https://cris.nih.go.kr/cris/search/detailSearch.do?seq=21448

## Introduction

Panic disorder is a common and important disease in clinical practice that leads to a reduction of individual productivity and increased use of health care [1]. The lifetime prevalence of panic disorder in the general population is 4.8%, and 22.7% of people experience panic attacks [2]. The most common symptoms of panic disorder include palpitations, shortness of breath, chest pain, numbness of the hands and feet, and cardiorespiratory-type symptoms, in addition to fear of dying, sweating, tremors, dizziness, nausea, and chills [3]. The US Food and Drug Administration has currently only approved selective serotonin reuptake inhibitors (SSRIs) for the treatment of panic disorder [4]. However, it is clinically difficult to expect an improvement in symptoms using SSRIs alone in the acute phase; thus we treat patients with benzodiazepine, which can lead to dependence and withdrawal symptoms [5,6]. The most common side effects of SSRIs reported by patients are reduced sexual function, drowsiness, and weight gain [7], and clinicians may hesitate to use benzodiazepines due to dependence and withdrawal symptoms [8]. Cognitive behavioral therapy (CBT) is the most widely used nonpharmaceutical treatment for anxiety disorders [9]. Additional nonpharmaceutical treatments, such as group therapy and supportive psychotherapy, are also available for patients with panic disorder [10,11]. However, these treatments have the disadvantage of requiring face-to-face contact; therefore, other therapeutic alternatives should be offered to patients during pandemics such as COVID-19.

The definition of a digital therapeutic (DTx) is a therapeutic that delivers evidence-based interventions to prevent, manage, or treat a medical disorder or disease; DTxs are currently used in many areas [12]. This kind of medical and public health use of smartphones and digital technologies is also known as mobile health (mHealth). DTxs related to mental health medicine are actively used in various psychiatric disorders, such as insomnia, substance abuse, attention-deficit/hyperactivity disorder, and anxiety and depression, among others [13]. In particular, the use of Freespira, a panic disorder DTx, reduced panic symptoms, avoidance behaviors, and treatment costs in patients with panic disorder [14].

As brain imaging technology advances, a great deal of functional mapping information on the human brain has been accumulated from positron emission tomography (PET), functional magnetic resonance imaging (fMRI), and functional near-infrared spectroscopy (fNIRS). Among these technologies, fNIRS can measure brain activity in a noninvasive and safe manner through measuring changes in the hemoglobin oxygenation state of the human brain [15]. Various studies have been conducted using fNIRS and fMRI to reveal correlations between panic disorder and brain regions. For example, patients with panic disorder show increased activity in the inferior frontal cortex, hippocampus, cingulate (both anterior and posterior), and orbitofrontal cortex (OFC) [16]. Previously, we confirmed that patients with panic disorder during rest periods showed increased activity in the OFC [17].

In this study, we determined whether an app for panic disorder would improve panic disorder symptoms. In addition, we used fNIRS to confirm the association between changes in panic disorder symptoms and changes in activity in specific brain regions.

## Methods

### Participants

Patients who had experiences of panic attacks were recruited between March 1 and July 30, 2022, through billboard advertisements at our hospital. The inclusion criteria for the study were as follows: (1) age between 20 and 65 years, (2) diagnosis of panic disorder based on the *Diagnostic and Statistical Manual of Mental Disorders, Fifth Edition* and (3) ability to use apps without problems. The exclusion criteria were as follows: (1) a history of other psychiatric disorders, except for anxiety disorder, or substance dependence, except for habitual alcohol and tobacco use; and (2) a history of head trauma and chronic medical conditions. The research clinician assessed whether patients fulfilled the inclusion or exclusion criteria. Written informed consent was acquired from all participants at the first visit. This study has been registered with the Clinical Research Information Service (KCT0007280).

### Assessment Scales for Anxiety Symptoms

The severity of panic symptoms was assessed using the Panic Disorder Severity Scale (PDSS). The PDSS was developed by Shear et al [18] in 1997. It is a 7-item instrument used to rate the overall severity of panic disorder and was validated in Korea by Lim et al [19] in 2001.

The anxiety symptoms of all participants were assessed using the clinician-based Hamilton Anxiety Scale (HAM-A) questionnaire and the participant-based Generalized Anxiety Disorder-7 (GAD-7) questionnaire. The HAM-A was developed by Hamilton in 1969 [20]. The 14-item version remains the most used outcome measure in clinical trials of treatments for anxiety disorders and was validated in Korea by Kim [21] in 2001.

The GAD-7 questionnaire, developed by Spitzer et al [22], is a 7-item self-report anxiety questionnaire designed to assess the patient’s health status during the previous 2 weeks. The GAD-7 was translated into the Korean language and is freely downloadable on the Patient Health Questionnaire website [23].

### Hemodynamic Response of the Prefrontal Cortex

The hemodynamics in the frontal cortex during the resting state were measured using the fNIRS device (NIRSIT; OBELAB Inc). The NIRSIT has 24 laser diodes (sources) emitting light at 2 wavelengths (780 nm and 850 nm) and 32 photodetectors with a sampling rate of 8.138 Hz [24]. The distance between the source and photodetector is 15 mm. Based on the suggested suitable sensor-detector separation distance for measuring cortical hemodynamic changes, only 30-mm channels were analyzed in this study [25].

**Figure 1:**
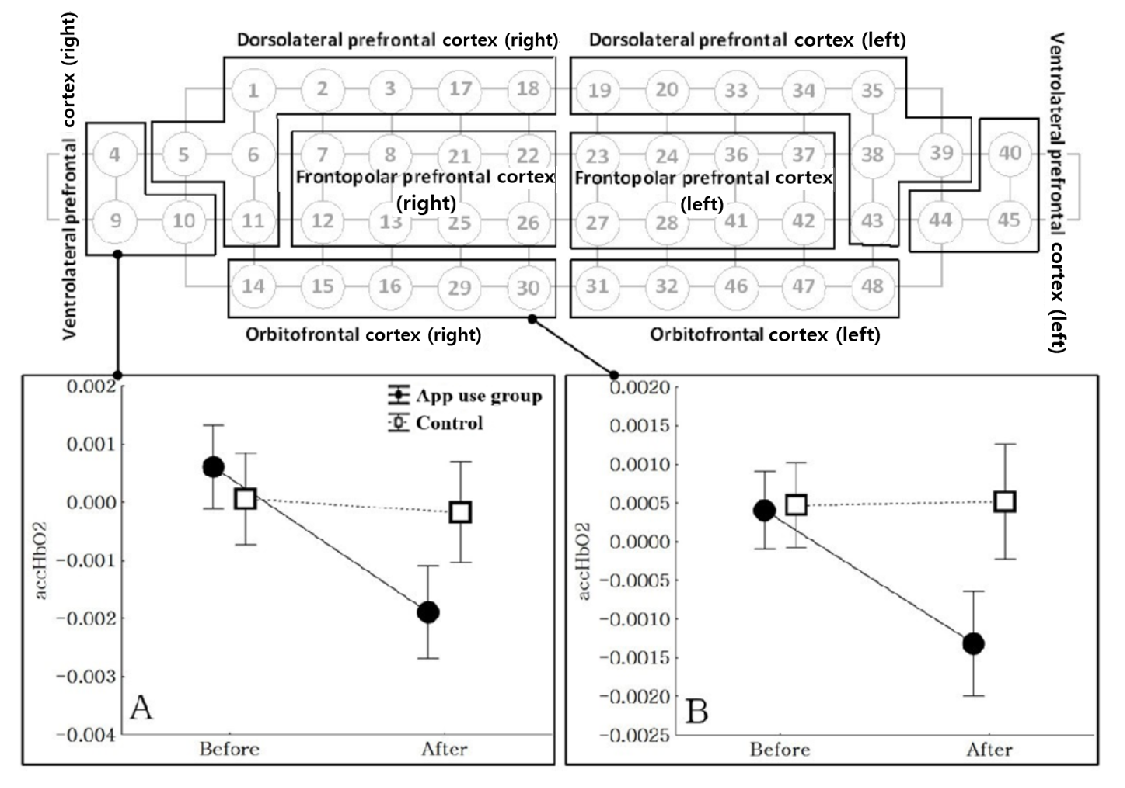
Illustration of the functional near-infrared spectroscopy channels and their respective regions, with the change in accumulated oxygenated hemoglobin (accHbO_2_) in each region. (A) Comparison of changes in accHbO_2_ in the right ventrolateral prefrontal cortex in the app use and control groups (F1,44=8.22; *P*=.006). (B) Comparison of changes in accHbO_2_ values in the right orbitofrontal cortex in the app use and control groups (F1,44=8.88, *P*=.005).

For our study, we used the 48-channel configuration (Figure 1). The detected light signals in each wavelength were filtered with a band-pass filter (0.00 Hz-0.1 Hz) to reduce the effect of environmental noise–related light and body movements. In addition, channels with low-quality information (signal-to-noise ratio <30 dB) were removed from the hemodynamic analysis. The accumulated oxygenated hemoglobin (accHbO_2_) values in the resting state represent the activation of the prefrontal cortex. In accordance with the theory that oxygenated hemoglobin has superior sensitivity and signal-to-noise ratio compared to deoxygenated hemoglobin data, only oxygenated hemoglobin were used for this analysis [26-28].

The means and SDs for accHbO_2_ were calculated from regions of interest (ROIs) in the right and left dorsolateral prefrontal cortices (DLPFCs), right and left ventrolateral prefrontal cortices (VLPFCs), right and left frontopolar cortices (FPCs), and right and left orbitofrontal cortices (OFCs), based on Brodmann area 46. The right and left DLPFCs comprise channels 1, 2, 3, 5, 6, 11, 17, and 18 and channels 19, 20, 33, 34, 35, 38, 39, and 43, respectively. The right and left VLPFCs comprise channels 4, 9, and 10 and channels 40, 44, and 45, respectively. The right and left FPCs comprise channels 7, 8, 12, 13, 21, 22, 25, and 26 and channels 23, 24, 27, 28, 36, 37, 41, and 42, respectively. The right and left OFCs comprise channels 14, 15, 16, 29, and 30 and channels 31, 32, 46, 47, and 48, respectively (Figure 1).

### Digital App for Panic Disorder

The app for panic disorder is a smartphone-based app for treatment of clinical symptoms of panic disorder. The mobile app has 4 categories: diary, education, quest, and serious games. The diary category has three items: (1) assessment of daily psychological status, including mood and anxiety; (2) assessment of panic symptoms, including frequency and severity; and (3) consumption of medication, including regular medication and pro re nata medications. The education category has three items: (1) knowledge about panic disorders, (2) knowledge about medications for panic disorder, and (3) knowledge about panic disorder treatment, including CBT, breathing therapy, and positive thinking therapy. The quests include two treatments: (1) eye movement desensitization and reprocessing therapy and (2) positive thinking therapy. The serious games include two games: (1) a breathing game and (2) an exposure therapy game.

The diary, education, and serious games (ie, the breathing game and exposure therapy game) are important parts of CBT for panic disorder [29-32]. The efficacy of CBT for panic disorder has been examined in various randomized controlled trials [33,34]. Eye movement desensitization and reprocessing therapy are also known to help reduce panic symptoms [35,36]. We confirmed that the replacement of worry with different forms of positive ideation shows beneficial effects [37], so a similar type of positive thinking therapy can also be expected to show benefits. Multimedia Appendix 1 provides additional information on the app.

### Ethical Considerations

The study trial was approved by the institutional review board of Chung-Ang University Hospital (1041078-202112-HR-349-01) and written informed consent was obtained from all participants. Participants received an explanation from the researchers that included an overview of the study and a description of the methodology and purpose before deciding to participate. Additionally, they were informed that participation was voluntary, informed about our confidentiality measures, given the option to withdraw, and informed about potential side effects and compensation. Participants in this study received ₩100,000 (US $75.50) as transportation reimbursement. Additionally, the various scales and fNIRS assessments were offered at no cost to the participants. The participants received the results of the tests in the form of a report via postal mail or email after the conclusion of the study. They also receive an explanatory document and consent form from the researchers that included contact information for any inquiries. If the participant agreed to take part in the study after understanding the consent form, the research proceeded. The participants’ personal information was not collected. Instead, a unique identifier was assigned to the collected data for the sole purpose of research management.

### Study Procedure

A randomized and treatment-as-usual–controlled design was applied in this study. After screening, all participants with panic disorder were randomly assigned to the app use group or the control group. The randomization sequence in our design was generated using SPSS (version 24.0; IBM Corp), with a 1:1 allocation between groups. At baseline and after intervention, all patients with panic disorder were assessed with the PDSS for panic symptoms, the HAM-A for objective anxiety symptoms, and the GAD-7 for subjective anxiety symptoms. At baseline and after intervention, the hemodynamic response in all patients with panic disorder was assessed using NIRSIT. The app use group was asked to use the app for panic disorder 20 minutes per day, 5 times per week, for 4 weeks. The control group was asked to read short educational letters that were delivered via a social network service 5 times per week for 4 weeks. The short letters contained information about panic disorder and its treatment.

## Results

### Demographic and Clinical Characteristics

**Figure 2:**
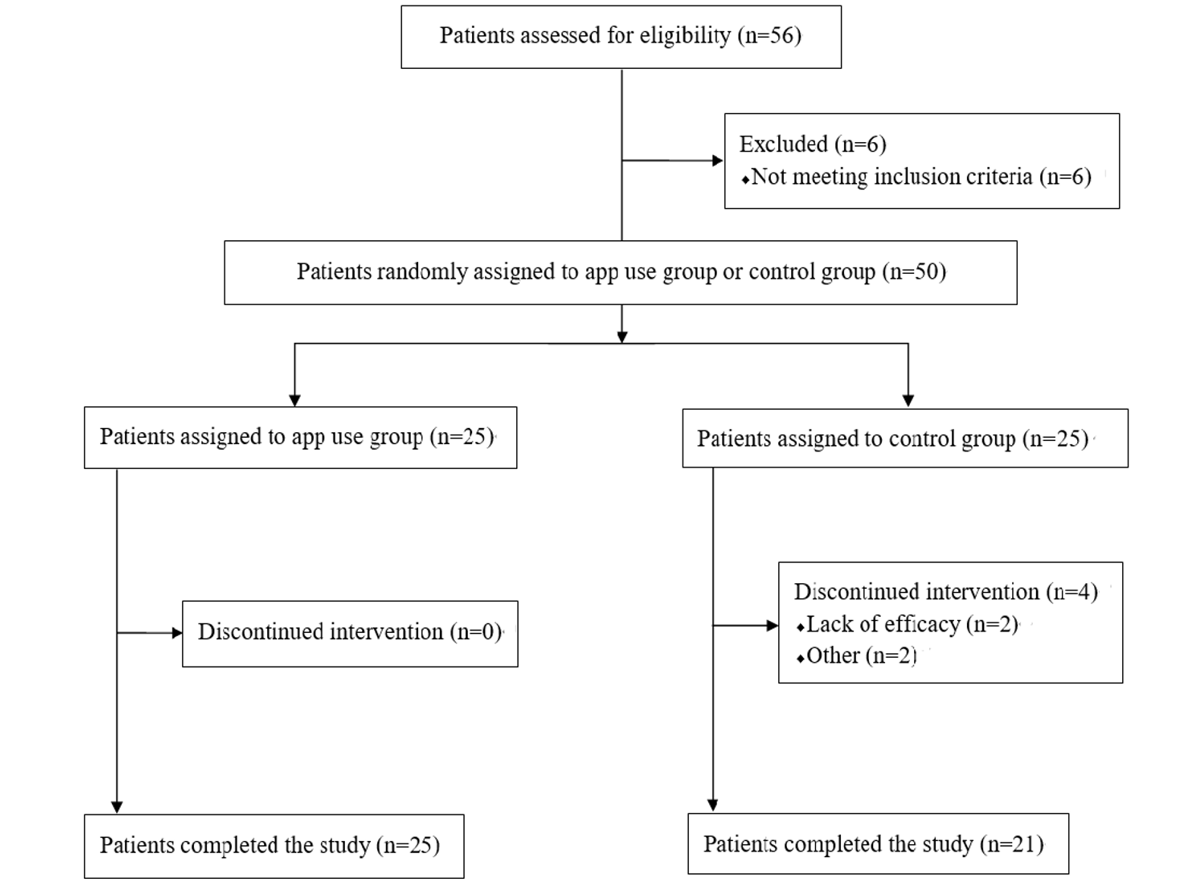
Consolidated Standards of Reporting Trials (CONSORT) flowchart.

After recruitment, 56 patients underwent eligibility assessments. A total of 6 individuals were excluded because they did not meet the inclusion criteria. The remaining patients were divided into 2 groups: 25 were assigned to the app use group and 21 to the control group, as 4 patients were excluded; contact was suddenly lost with 1 patient contact and 1 dropped out for personal reasons. In addition, 2 patients in the control group quit the study after reporting poor benefits from the short educational letters. Therefore, 25 people in the app use group and 21 people in the control group were analyzed. Figure 2 shows the Consolidated Standards of Reporting Trials (CONSORT) flowchart for participant flow through the trial.

There were no significant differences in age, sex ratio, years of education, marital status, employment status, or substance habits, including smoking and alcohol use, between the app use group and the control group (Table 1).

**Table 1:** Demographic characteristics. There were no significant differences in demographic characteristics between the app and control groups.

		App use group (n=25)	Control group (n=21)	*t* test (*df*) or chi-square (*df*)	*P* value	
Age (years), mean (SD)		36.5 (12)	33.1 (12)	0.97 (45)^a^	.33	
**Sex, n (%)**						
	Male	4 (16)	8 (38)			
	Female	21 (84	13 (62)	2.89 (1)^b^	.11	
**Education, n (%)**						
	High school	5 (20)	9 (43)	5.50 (1)^b^	.06	
	University	17 (68)	7 (33)			
	Undergraduate	3 (12)	5 (24)			
**Marital status, n (%)**						
	Married	10 (40)	9 (43)	0.07 (1)^b^	.97	
	Not married	14 (56)	11 (52)			
	Divorced	1 (4)	1 (5)			
**Employment status, n (%)**						
	Unemployed	2 (8)	5 (24)	3.07 (1)^b^	.38	
	Student	5 (20)	4 (19)			
	Part-time employment	6 (24)	6 (29)			
	Regular employment	12 (48)	6 (29)			
**Smoking, n (%)**						
	<10 cigarettes/month	23 (92)	17 (81)	2.59 (1)^b^	.46	
	10-19 cigarettes/month	1 (4)	2 (10)			
	>20 cigarettes/month	1 (4)	2 (10)			
**Alcohol, n (%)**						
	≤1 time/month	15 (60)	14 (67)			
	2-4 times/month	9 (36)	7 (33)	1.30 (1)^b^	.73	
	≥4 times/month	1 (4)	0 (0)			
**Clinical scores, mean (SD)**						
	Hamilton Anxiety Scale	22.7 (4.1)	21.8 (5.6)	0.66 (45)^a^	.51	
	Generalized Anxiety Disorder–7	10.4 (4.7)	8.9 (5.4)	1.00 (45)^a^	.32	
	Panic Disorder Severity Scale	14.3 (6.7)	12.4 (6.2)	1.05 (45)^a^	.29	

^a^*t* test.

^b^Chi-square.

There were no significant differences in HAM-A score, GAD-7 score, or PDSS score at baseline between the app use group and control group (Table 1).

### Comparison of Changes in Clinical Scales Between App Use Group and Control Group

The number of participants with improved panic symptoms in the app use group (20/25, 80%) was greater than in the control group (6/21, 29%; *χ*^2^_1_=12.3; *P*=.005).

**Figure 3:**
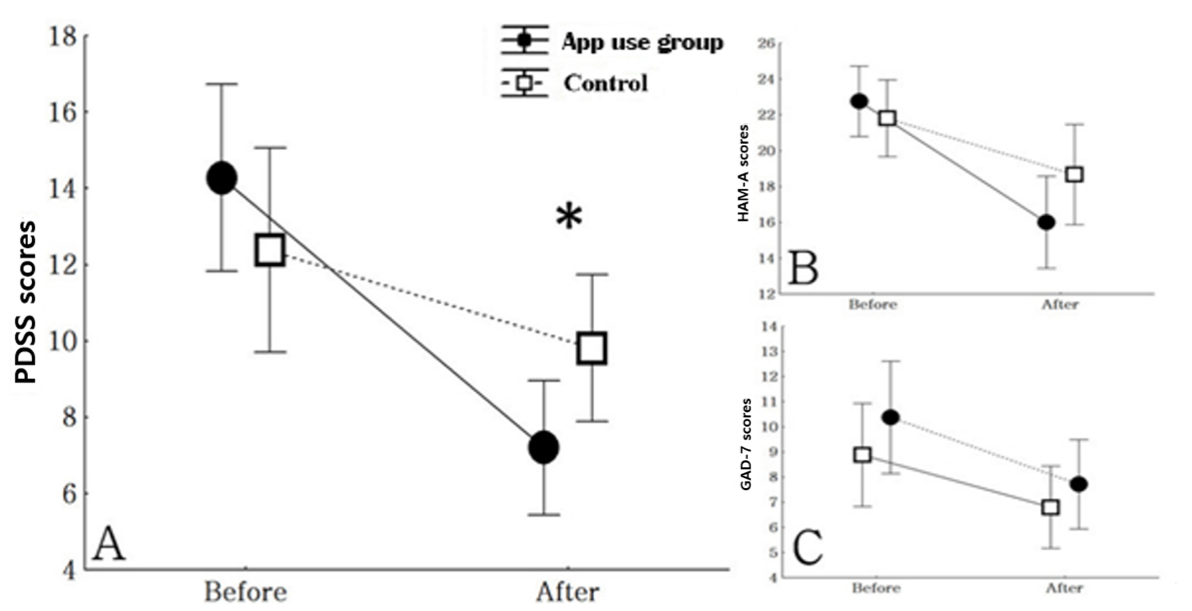
Illustration of changes in the (A) Panic Disorder Severity Scale (PDSS), (B) Hamilton Anxiety Scale (HAM-A), and (C) Generalized Anxiety Disorder–7 (GAD-7) scores before and after app use. The comparison used a repeated measures ANOVA. *P<.05.

During the treatment period, the app use group showed greater improvement in PDSS score than the control group (*F*_1,44_=7.03; *P*=.01). In the app use group, the PDSS score decreased by 42.5% (mean score 14.3, SD 6.5 at baseline and mean score 7.2, SD 3.6 after the intervention), while the score decreased by 14.6% in the control group (mean score 12.4, SD 5.2 at baseline and mean score 9.8, SD 7.9 after intervention) (Figure 3).

During the treatment period, there were no significant differences in the change in HAM-A scores (*F*_1,44_=2.83; *P*=.09) and GAD-7 scores (*F*_1,44_=0.22; *P*=.64) between the app use group and control group (Figure 3).

### Comparison of Changes in accHbO_2_ Values Between App Use Group and Control Group

There were no significant differences in accHbO_2_ in the right (t_45_=0.84; *P*=.40) or left (t_45_=0.73; *P*=.46) DLPFCs, right (t_45_=1.04; *P*=.31) or left (t_45_=0.88; *P*=.39) VLPFCs, right (t_45_=-0.18; *P*=.86) or left (t_45_=1.85; *P*=.07) FPCs, or right (t_45_=0.33; *P*=.74) or left (t_45_=1.89; *P*=.07) OFCs in the app use and control groups at baseline.

During the treatment period, the app use group showed a greater reduction in accHbO_2_ in the right VLPFC (*F*_1,44_=8.22; *P*=.006) and right OFC (*F*_1,44_=8.88; *P*=.005) compared to the control group (Figure 1). During the treatment period, there were no significant differences in the change in accHbO_2_ in the other ROIs between the app use and control groups.

### Correlations Between the Changes in PDSS Scores and the Changes in accHbO_2_

In all participants (ie, the app use group plus the control group), there was a positive correlation between the change in PDSS score and the change in accHbO_2_ in the right VLPFC (*r*=0.44; *P*=.002). In the app use group, there was a positive correlation between the change in PDSS score and the changes in accHbO_2_ in the right VLPFC (*r*=0.42; *P*=.04). However, in the control group, there was no significant correlation between the change in PDSS score and the change in accHbO_2_ in the right VLPFC (*r*=0.22; *P*=.16).

**Figure 4:**
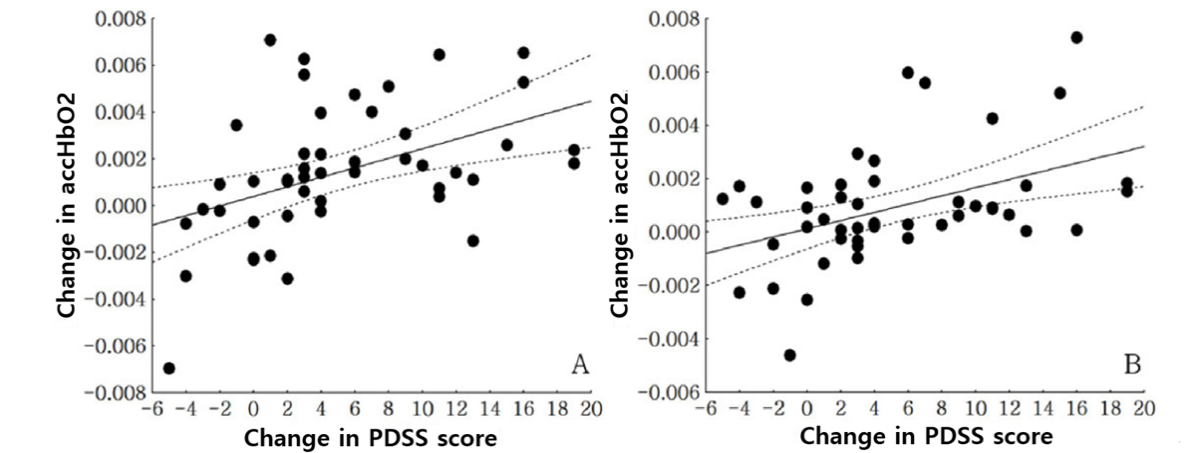
Illustration of the correlation between changes in (A) the Panic Disorder Severity Scale (PDSS) score and (B) accumulated glycated hemoglobin (accHbO_2_) variations across different brain regions.

In all participants, there was a positive correlation between the change in PDSS score and the change in accHbO_2_ in the right OFC (*r*=0.44; *P*=.002). In both the app use group (*r*=0.34; *P*=.09) and control group (*r*=0.33; *P*=.13), there was no significant correlation between the change in PDSS score and the change in accHbO_2_ in the right OFC (Figure 4).

## Discussion

### Principal Findings

This study showed that a digital app was effective for symptom reduction, as well as decreasing brain activity in the VLPFCs and OFCs, in patients with panic disorder. In addition, the panic disorder symptom improvement was positively correlated with decreased brain activity in the VLPFCs and OFCs in the resting state.

The digital app used in this trial proved to be effective in reducing panic symptoms when compared to the control group, as demonstrated by the reduction in the PDSS score. We believe that this is due to the combined effect of the 4 parts of the program, namely the diary, education, quest, and serious games. The diary component helps identify and correct faulty perceptions and enables cognitive reconstruction. The education component provides information about the nature and physiology of panic disorder. The breathing game helps the participant return to a relaxed condition, while the exposure therapy game allows the participant to experience agoraphobic situations in a safe environment, which helps cognitive restructuring. These are the important parts of CBT for panic disorder and have shown efficacy, as reported earlier [29-32]. The control group also received educational data, including the importance of keeping a diary of one’s panic symptoms and how to do it, as well as self-guided direction on breathing exercises, but failed to show a significant reduction of symptoms compared to the app use group. We think this is due to lack of proper feedback in the control group. The app shows real-time feedback on breathing exercises using breathing sounds, and a message was sent if the user of the program failed to use the program for more than 2 days. We know that the therapeutic effect is better when immediate feedback is provided to patients undergoing CBT treatment [38]. Therefore, we think that the decrease in PDSS score was smaller because the control group did not receive feedback from the app.

The control group also received educational data on diary recording, panic disorder information, and how to execute breathing therapy and exposure therapy. We measured their reduction in the PDSS score, but we found it was less than in the app use group due to a lack of proper daily management.

However, the app failed to lead to a difference in the reduction in anxiety, as defined by the HAM-A and GAD-7 scales, between the 2 groups. This is most likely due to a lack of power, as the trial was conducted as a pilot study. Other studies using CBT techniques or serious games have demonstrated reductions in anxiety symptoms in patients with panic disorder [14]. Likewise, this study showed a trend toward a reduction in anxiety symptoms, although this was not statistically significant, and future research with more participants may show that these kinds of programs are also effective in controlling anxiety.

Two major changes in brain activity were noted in the app use group, namely reductions in VLPFC and OFC activation. The functions of the OFC are varied and include control of inappropriate behavior and emotional responses, decision-making, and solving problems [39,40]. Abnormalities in the function of the OFC can cause problems in dealing with anxiety and show that it is deeply involved in the increasing the sense of fear in the fear response [17]. The results of this study confirm that OFC activity decreases as treatment progresses. This reinforces the results of a previous study, which showed that patients with panic disorder had increased OFC activity and that when the panic disorder was treated, the activity of the OFC was reduced, as indicated by decreased cerebral glucose metabolic rates [17,41].

The VLPFC is known to be associated with the amygdala and to maintain flexible attention and responses to environmental threats [42,43]. The amygdala is the backbone of the fear network, and the VLPFC is also known to be deeply involved in the processing of fear [43-45]. Several studies have shown increased activity in patients with panic disorder in the inferior frontal gyrus, which envelops the VLPFC, and other related regions, including the prefrontal cortex, hippocampus, and OFC [16,46,47]. After panic disorder treatment, such as with CBT, decreased amygdala and inferior frontal gyrus activation in fear situations was confirmed [48,49]. Through panic disorder treatment, inferior frontal gyrus activation decreased to a normal level; this happened because the treatment reduced fear cognition related to harm expectancy or attention to threats [49-51]. We consider that VLPFC activation increases to modulate the amygdala and decreases with treatment for panic disorder.

We believe that these reductions of brain activity in the VLPFC and OFC reflect how the app affected the patients. We know that overprediction of fear or panic is an important feature of anxiety disorders [52]. The app for panic disorder, including diary, education, quest, and serious game components, allowed users to correct their faulty perceptions about fear. As mentioned earlier, the VLPFC and OFC are related to fear management, so we can expect that activity of the VLPFC and OFC will be reduced through repeated app use as users learn how to deal with fear.

### Limitations

This study has the following limitations: Most of the patients were effectively treated with alprazolam or other anxiolytics, such as SSRIs. Thus, treatment with antianxiety drugs may have influenced our results. Moreover, this study assessed changes immediately after app use. A long-term follow-up to evaluate the sustainability of the observed improvements would provide valuable insights into the effectiveness of the intervention over time. App use time could be easily tracked for the app use group; however, it was challenging to independently monitor the time the control group spent reading educational materials. Due to the limitations of available research tools, no investigation has been conducted on deep brain structures such as the amygdala, which is most closely related to panic disorders.

### Conclusions

We believe that this app for panic disorder effectively reduces symptoms and noticeably impacts brain activity in specific areas. We observed a positive link between improvement in panic symptoms and decreased brain activity in the VLPFCs and OFCs in a resting state. These findings support the use of targeted interventions to determine the brain’s contribution to symptom relief. Further research should explore the duration of these positive effects and make digital therapy accessible to more individuals, thus unlocking its full potential in mental health care.

## Appendix

Multimedia Appendix 1 Digital app for panic disorder.

Multimedia Appendix 2 CONSORT-eHEALTH checklist (V 1.6.1).

## Abbreviations

accHbO_2_: accumulated oxygenated hemoglobin
CBT: cognitive behavioral therapy
DLPFC: dorsolateral prefrontal cortex
DTx: digital therapeutic
fNIRS: functional near-infrared spectroscopy
FPC: frontopolar cortex
GAD-7: Generalized Anxiety Disorder–7
HAM-A: Hamilton Anxiety Scale
mHealth: mobile health
OFC: orbitofrontal cortex
PDSS: Panic Disorder Severity Scale
ROI: region of interest
SSRI: selective serotonin reuptake inhibitor
VLPFC: ventrolateral prefrontal cortex

## Data Availability

The data sets generated and analyzed during this study are not publicly available as they contain information that could compromise the privacy and consent of the research participants. However, the transformed data are available upon reasonable request from the authors.
