# Supplementary material for: The Effectiveness of a Digital App for Reduction of Clinical Symptoms in Individuals With Panic Disorder: Randomized Controlled Trial
Source: J Med Internet Res. 2024 Apr 12;26:e51428. doi: 10.2196/51428 (PMC11053392; doi:10.2196/51428)
Supplement: Multimedia Appendix 1 [file jmir_v26i1e51428_app1.pdf]

Supplement 1. Digital app for panic disorder, (Diary)

A)

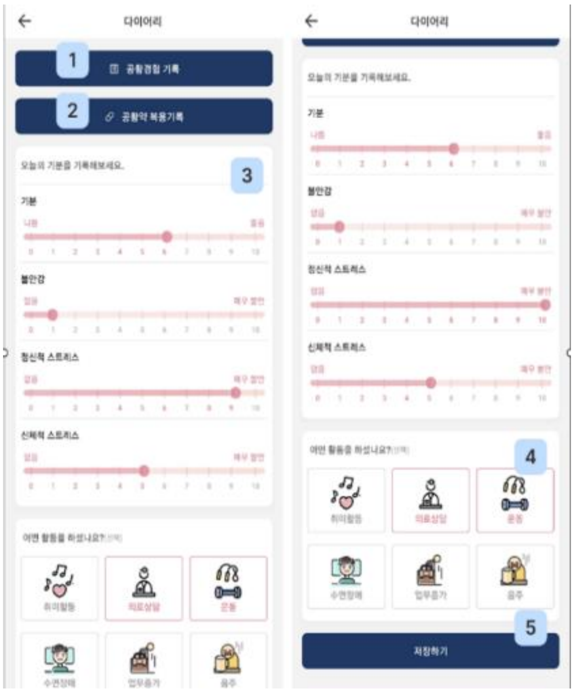

B)

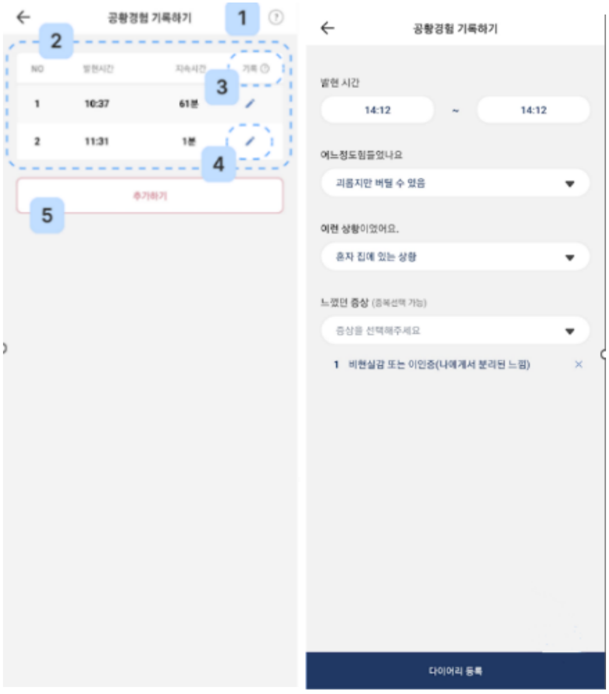

C)

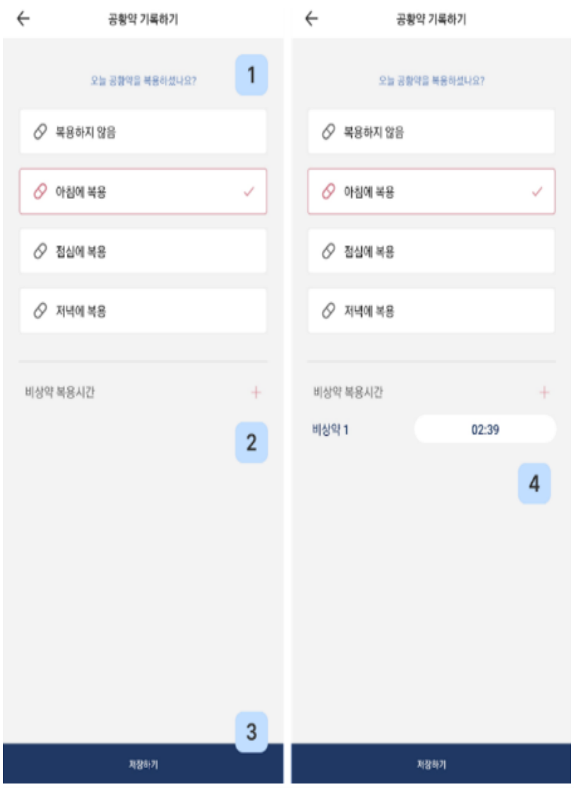

Screenshots of Digital app for panic disorder

A) Diary Main (Diary)

Diary Main screen, Users can save their diaries after registering all four tasks: panic recording, panic medication recording, today's mood and activity

B) Panic experience record (Diary)

The user can record occurrence and duration time of panic symptoms. The user can set how hard or difficult the panic was and can choose which situation he experienced panic in. The user chooses what symptoms he felt during the panic and filled out all his entries, he can register his diary.

C) Panic medication record (Diary)

The user can choose when he took the panic pill. The user can add time to take emergency medication.

Supplement 2. Digital app for panic disorder, (Quest)

A)

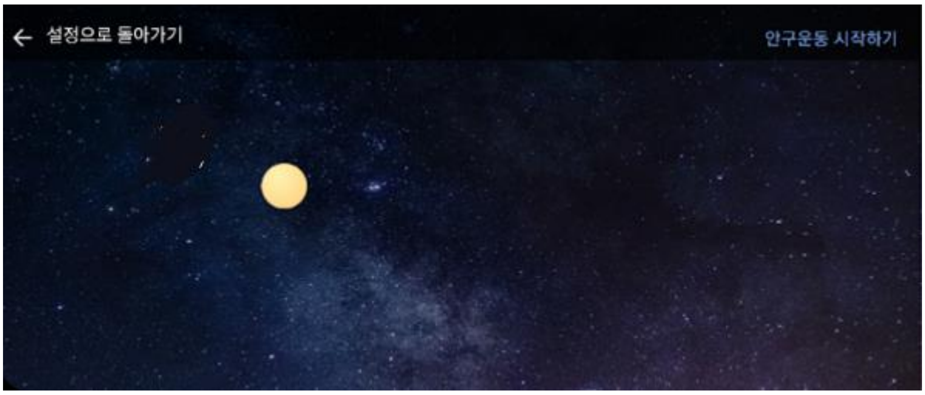

B)

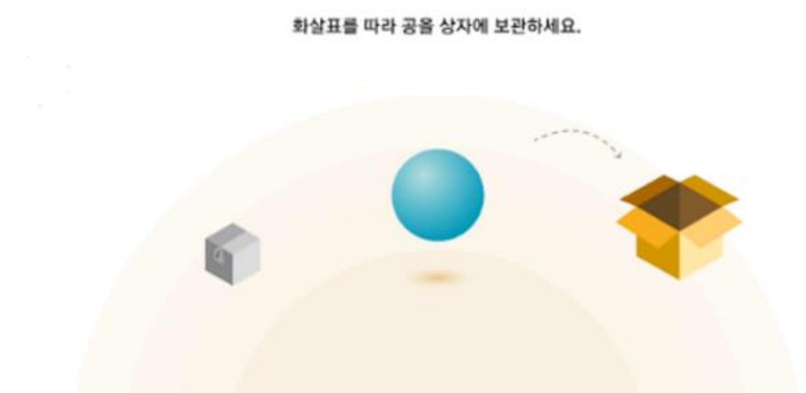

Screenshots of Digital app for panic disorder

A) Eye movement desensitization and reprocessing therapy (Quest)

The user hold cell phone horizontally and look at the screen. This quest relieve anxiety through eye movement by imagining not only memories of panic but also painful experiences. The user can change the color, size, and speed of the circle. The user performs quest after viewing an video that causes anxiety.

B) Positive thinking therapy (Quest)

There is gray box on the screen that can throw away negative thoughts and yellow box that allow you to store positive thoughts. The user bounces the ball and moves the negative thoughts to the gray box and the positive thoughts to the yellow box.

Supplement 3. Digital app for panic disorder, (Serious game)

A)

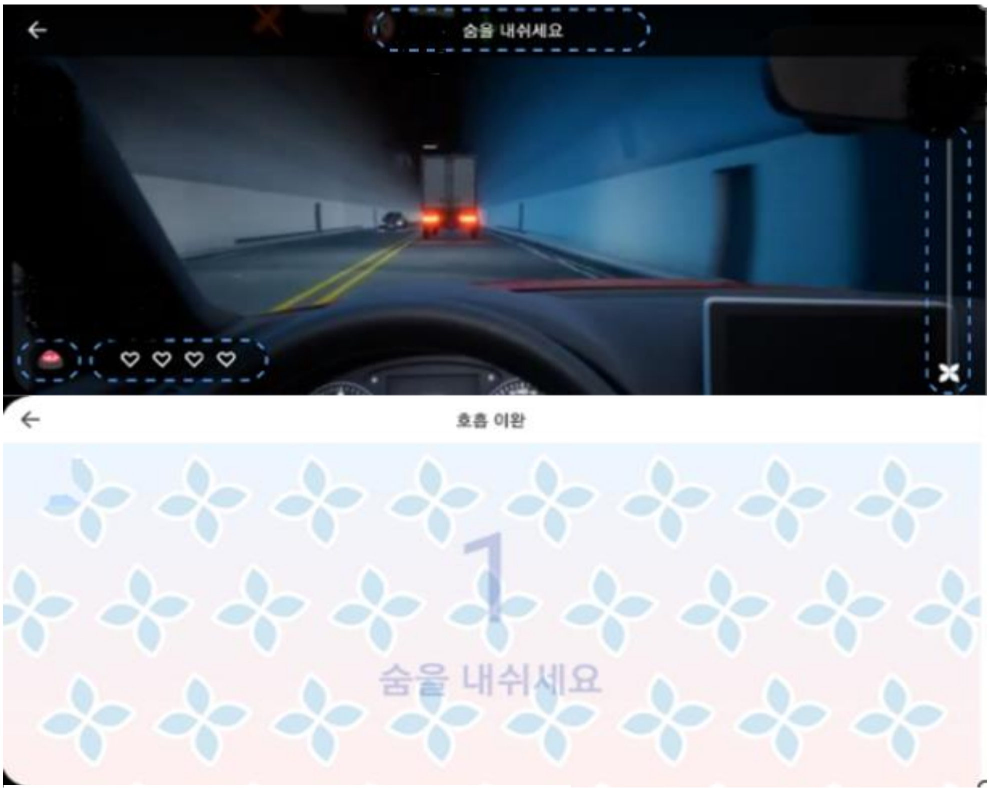

B)

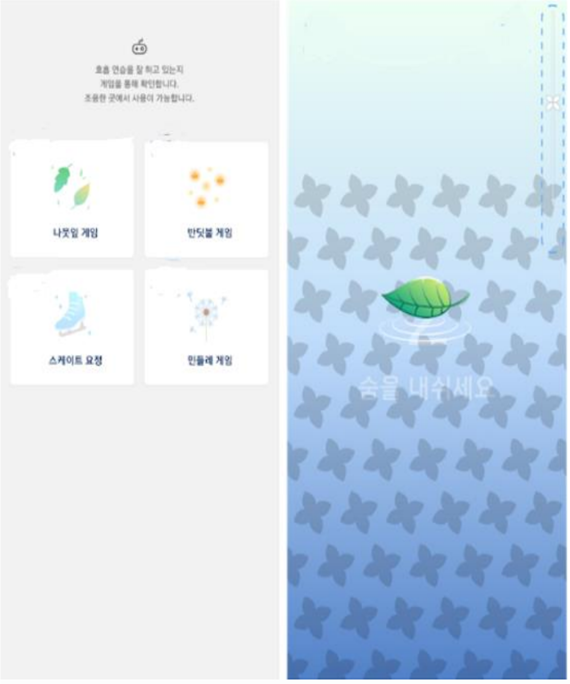

Screenshots of Digital app for panic disorder

A) Exposure therapy (Serious game)

There are five situations that can cause panic (in the car driving through tunnels, underground tunnels, subways, announcements in front of spectators, and stuffy rooms). The user can choose one situation and start the game. Each minute after the start of the game, the heart at the bottom left of the screen will be filled one space each,

and the game will be completed after a total of four minutes. If you want to stop in the middle of the game or after the game is completed, it will lead to a breath relaxation therapy screen.

B) Breathing games (Serious game)

The user inhales as much as the countdown time, holds his or her breath, and then exhales again. Images 1 to 4 Each game has a different design, which helps maintain breathing tension as the image on the screen moves during the breathing time. This is repeated for a certain amount of time. Images on the screen move during breathing time, helping to maintain breathing tension.

Study participant flowchart – CONSORT diagram

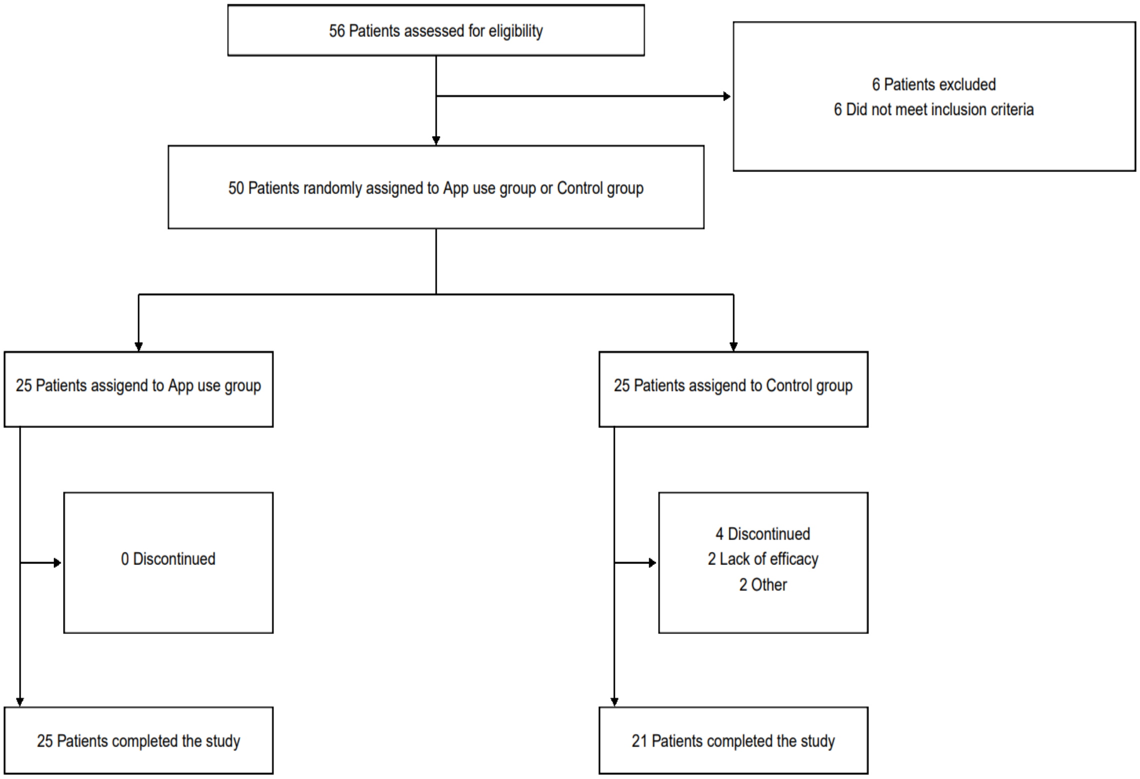

Between March 1st and July 30th, 2022, we recruited individuals experiencing panic attacks through billboard ads at Chung-ang University. Out of 56 patients assessed, 6 were excluded. Following a randomized and treatment-controlled design, participants with panic disorder were randomly assigned to either the App use or Control group (25 participants each). 4 people were eliminated from the Control group. One was suddenly lost the contact and one was dropped for personal reasons. In addition, the two quit because complained about poor benefit from the educational data which combined with Control group. The App group used panic disorder Apps for 20 minutes/day, 5 times/week for 4 weeks, while the Control group read informative letters about panic disorder and its treatment.
